# Lifestyles associated with complete tooth loss in elderly people in Brazil, 2019

**DOI:** 10.1590/S2237-96222025v34e20240614.en

**Published:** 2025-05-12

**Authors:** Zamir Vidal de Negreiros, Nizyara Costa da Silva, Rafaela Alcindo Silva de Sousa Fé, Mariella Agostinho Gonçalves Lourenço, Rafael Barroso Pazinatto, Ana Estéfanny Alves Cabral, Laércio Almeida de Melo

**Affiliations:** 1 Universidade Federal do Rio Grande do Norte, Departamento de Odontologia, Natal, RN, Brazil; 2 Universidade Federal de Juiz de Fora, Departamento de Odontologia Restauradora, Juiz de Fora, MG, Brazil

**Keywords:** Elderly, Lifesyle, Tooth Loss, Precipitating Factors, Cross-Sectional Studies, Anciano, Estilo de Vida, Pérdida de Diente, Factores Desencadenantes, Estudios Transversales

## Abstract

**Objective:**

This study aimed to assess types of lifestyle that may contribute to total tooth loss in the elderly.

**Methods:**

This is a cross-sectional and population-based study, having as its target population elderly individuals aged 60 years or older. We used the database of the most recent edition of the National Health Survey, conducted in Brazil in 2019. Initially, the chi-square test was used and then the prevalence ratios were adjusted using the Poisson multiple regression model in order to identify associations between the variables.

**Results:**

The final sample analyzed consisted of 22,728 elderly individuals. Prevalence of complete tooth loss was 31.7% (95% confidence interval [95%CI] 31.1; 32.3). Multivariate analysis revealed that this condition was higher in females (p-value<0.001; prevalence ratio [PR] 1.05; 95%CI 1.04; 1.07), in the oldest old (p-value<0.001; PR 1.54; 95%CI 1.43; 1.61), in those without formal education (p-value<0.001; PR 1.06; 95%CI 1.04; 1.08), in those without dental insurance (p-value<0.001; PR 1.07; 95%CI 1.05; 1.09), in smokers (p-value<0.001; PR 1.04; 95%CI 1.02; 1.06), in individuals who consume soft drinks with high sugar content (p-value<0.001; PR 1.05; 95%CI 1.03; 1.07) and in those who do not do physical activities (p-value<0.001; PR 1.05; 95%CI 1.03; 1.06).

**Conclusion:**

We concluded that complete tooth loss was greater in elderly people with unfavorable socioeconomic conditions, those who smoke, those who consume soft drinks with high sugar content and those who do not do physical activities regularly.

## Resumen

**Objetivo:**

Este estudio tuvo como objetivo evaluar los tipos de estilo de vida que pueden contribuir a la pérdida total de dientes en las personas mayores.

**Métodos:**

Este estudio se caracteriza por ser transversal y poblacional, siendo la población objetivo personas mayores de 60 años o más. Se utilizó la base de datos de la última edición de la Encuesta Nacional de Salud realizada en Brasil, en 2019. Inicialmente se utilizó la prueba de chi cuadrado y luego se ajustaron las razones de prevalencia mediante el modelo de regresión múltiple de Poisson con el objetivo de identificar asociaciones entre las variables.

**Resultados:**

La muestra final analizada fue de 22.728 personas mayores. La prevalencia de pérdida dental completa fue del 31,7% (intervalo de confianza del 95% [IC95%] 31,1; 32,3). El análisis multivariado reveló que esta condición era mayor en el sexo femenino (p-valor<0,001; razón de prevalencia [RP] 1,05; IC95% 1,04; 1,07), en ancianos más viejos (p-valor<0,001; RP 1,54; IC95% 1,43; 1,61), en aquellos sin educación (p-valor<0,001; RP 1,06; IC95% 1,04; 1,08), en quienes no tienen seguro dental (p-valor<0,001; PR 1,07; IC95% 1,05; 1,09), en fumadores (p-valor<0,001; PR 1,04; IC95% 1,02; 1,06), en individuos que consumen refrescos con alto contenido de azúcar (p-valor<0,001; RP 1,05; IC95% 1,03; 1,07) y en aquellos que no practican actividad física (p-valor<0,001; RP 1,05; IC95% 1,03; 1,06).

**Conclusión:**

Se concluye que la pérdida dental completa fue mayor en personas mayores con condiciones socioeconómicas desfavorables, en quienes fuman, en quienes consumen refrescos con alto contenido de azúcar y en quienes no realizan actividad física regularmente.

## Introduction

In recent decades, prevalence of complete tooth loss has decreased in younger populations, but it remains prevalent in the elderly (1). Oral diseases still affect many elderly people, whereby dental caries and progression of periodontal disease are the main factors that influence tooth loss. This can contribute to functional problems, such as chewing and phonation. Aesthetic and social problems can also arise, affecting health and quality of life, especially when tooth loss is complete (2). Tooth loss has a multifactorial etiology and involves biological, physical, economic, cultural, social and even behavioral factors (3-6). 

The most frequent results found in the literature on factors associated with complete or partial tooth loss are those related to oral health (7). It is important to highlight that systemic factors, such as chronic non-communicable diseases, frailty and motor disability, can be important variables in the decline of oral health, which can consequently contribute to tooth loss (8-11). Habits such as smoking, drinking alcohol and poor lifestyles can also contribute to poor oral health. These variables are not included in most studies on tooth loss.

Nicotine in tobacco can contribute to the formation of cariogenic biofilm, as well as changes in the composition and quantity of saliva (12-15). In addition to influencing dental caries incidence, tobacco is a risk factor with regard to periodontal disease. Periodontal tissues become more inflamed among smokers, which contributes to increased risk of periodontitis developing and worsening (16,17). 

Salivary changes and damage to soft tissues in the mouth may be evident in individuals who have the habit of drinking alcohol. Although there is no direct relationship with complete tooth loss, unhealthy lifestyles, such as lack of physical exercise, contribute to functional and systemic deterioration of the elderly and can influence oral health (18,19). 

Given the accelerated aging of the global population and the consequences related to tooth loss, the objective of this study was to assess types of lifestyle that can contribute to complete tooth loss in the elderly. The null hypothesis of this study is that complete tooth loss is not greater among elderly people with poorer habits and unhealthy lifestyles.

## Methods

### Design

This is a cross-sectional, observational, population-based study. This study used the database of the National Health Survey conducted in Brazil in 2019. 

### Setting

The 2019 National Health Survey was conducted by the Brazilian Institute of Geography and Statistics in partnership with the Ministry of Health. The survey aimed to provide updated data on the health of the Brazilian population. The target population was comprised of people aged 15 or over and covered aspects such as health conditions, lifestyle, access to health services, morbidity, use of medication and mental health, in addition to outlining the population’s living conditions, with emphasis on regional and socioeconomic inequalities. Data collection took place between August 2019 and March 2020.

### Participants

This study analyzed data on individuals aged 60 years or older who provided information on the presence or absence of complete tooth loss in both the upper and the lower jaws. The National Health Survey sample is representative of Brazil’s elderly population. Details on the sample calculation can be found in the methods section of the National Health Survey (20).

### Independent variables

The independent variables, which have a possible influence on the complete tooth loss outcome, were collected via the questionnaire used in the National Health Survey. These variables are: sex, age, race/skin color, schooling, tooth brushing frequency, dental insurance, smoking, alcohol consumption, physical exercise, eating sweet biscuits or chocolate, eating quick snacks instead of having lunch and type of soft drink consumed.

### Complete tooth loss

Identification of individuals who presented complete upper and lower jaw tooth loss took place by analyzing the following questions contained in the National Health Survey: “Have you lost any of your upper permanent teeth?” and “Have you lost any of your lower permanent teeth?”. Answer options to these questions were: “No”, “Yes, I have lost XX teeth”, where “XX” represented the number of teeth lost, “Yes, I have lost all my upper teeth” and “Yes, I have lost all my lower teeth”. The “Complete tooth loss” variable was categorized as “present”, when all upper and lower teeth were lost, or “absent”, when there was no complete loss of teeth.

### Data source and measurement

This work used secondary data from the 2019 National Health Survey, conducted in Brazil. The data can be accessed from the following source: https://www.pns.icict.fiocruz.br/bases-de-dados/ (21).

### Bias control

Bias control in this study was based on the sampling approach used by the National Health Survey, which took into consideration the different selection probabilities and the characteristics of the complex sampling design. Sampling weights were assigned to both the households and the selected individuals. The final weighting was determined by inverse selection probabilities at each stage of the sampling plan, with adjustments for non-response and corrections of the population totals. Due to the sample originating from a cluster survey, specialized statistical analysis software was used, which took into account the effects of stratification and data aggregation in estimating the indicators and determining the respective accuracy measurements.

### Study size

The National Health Survey was conducted throughout Brazil. It covered all the country’s macro-regions, including urban and rural areas, involving households in state capital cities and metropolitan regions. Through its comprehensiveness and rigorous methodology, the survey provides a complete overview of the health conditions of the Brazilian population.

### Statistical methods

Data analysis was performed using the Statistical Package for the Social Sciences, version 22.0. Frequency distributions of the study variables were calculated and expressed in tables. We used the chi-square test with a 95% confidence level to assess association between complete tooth loss and the independent variables. Based on the results of this test, the chi-square test was applied between variables with a p-value<0.200 in order to test for multicollinearity. Variables that were highly related to each other (multicollinear) were not included in the adjusted model. Due to the study sample size, variables were considered multicollinear when their p-value<0.001. Adjusted prevalence ratios were estimated using Poisson multiple regression. The data were weighted in all tests, always taking into consideration the effect of the sampling plan, post-stratification weights, and non-response rates. A 95% confidence level was also used in the Poisson multiple regression.

## Results

This study’s sample consisted of 22,728 elderly individuals, with a mean age of 70.0 years (±7.8), ranging from 60-107 years. Frequency analysis of the results showed that the majority of the completely edentulous elderly individuals living in Brazil are female (62.7%), aged 60-69 years (40.5%), White (40.4%), literate (65.3%), without dental insurance (96.0%), who do not drink alcohol (84.7%), do not smoke (86.1%), brush their teeth at least once a day (99.1%), do not consume sweet cookies or chocolates during the week (59.9%), do not replace lunch with quick snacks on any day of the week (91.7%), consume sugar-sweetened soft drinks (94.3%) and do not undertake any type of physical activity (79.7%).

Prevalence of completely edentulous elderly individuals was 31.7% (95% confidence interval [95%CI] 31.1; 32.3). The frequency of the independent variables (socioeconomic and lifestyle variables), as well as their associations with complete tooth loss through univariate analysis are presented in Table 1. As a result of this first analysis and multicollinearity being found between the variables, “race/skin color”, “alcoholic beverage”, “tooth brushing frequency”, “eating sweet biscuits or chocolate” and “eating quick snacks instead of having lunch” were not included in the Poisson multiple regression adjustment model due to strong association with the other independent variables.

**Table 1:** Crude complete tooth loss prevalence ratios (PR) and 95% confidence intervals (95%CI), according to socioeconomic and lifestyle variables. Brazil, 2019 (n=22,728)

	Complete tooth loss present n=7,195	Complete tooth loss absent n=15,533		
Variable	n (%)	n (%)	PR (95%CI)	p-value
Sex				
Female	4.511 (36.0)	8.024 (64.0)	1.40 (1.32; 1.48)	<0.001
Male	2.684 (26.3)	7.509 (73.7)		
**Age** (years)				
60-69	2.917 (23.2)	9.638 (76.8)	1.00	<0.001
70-79	2.736 (38.2)	4.421 (61.8)	1.26 (1.23; 1.31)	
≥80	1.542 (51.1)	1.474 (48.9)	1.62 (1.53; 1.72)	
**Race/skin color^a^**				
White	2.906 (29.4)	6.995 (70.6)	1.00	<0.001
Mixed race	3.399 (34.0)	6.602 (66.0)	1.07 (1.04; 1.11)	
Black	779 (31.7)	1.676 (68.3)	1.06 (1.01; 1.12)	
Other	110 (29.8)	259 (70.2)	0.96 (0.87; 1.05)	
Schooling				
Illiterate	2.496 (46.4)	2.887 (53.6)	1.75 (1.65; 1.86)	<0.001
Literate	4.699 (27.1)	12.646 (72.9)		
**Dental insurance**				
No	6.910 (33.0)	14.018 (67.0)	2.09 (1.76; 2.48)	
Yes	285 (15.8)	1.515 (84.2)		
**Alcoholic beverage**				
Yes	1.102 (19.6)	4.530 (80.4)	0.51 (0.46; 0.56)	<0.001
No	6.093 (35.6)	11.003 (64.4)		
**Tobacco smoking**				
Yes	1.002 (37.4)	1.678 (62.6)	1.24 (1.14; 1.35)	<0.001
No	6.193 (30.9)	13.855 (69.1)		
**Tooth brushing frequency^a^**				
Not brushing teeth daily	61 (48.0)	66 (52.0)	1.32 (1.00; 1.75)	0.074
At least once a day	6.375 (29.7)	15.139 (70.4)		
**Eating sweet biscuits or chocolate**				
Yes	4.307 (32.6)	8.922 (67.4)	1.10 (1.04; 1.17)	<0.001
No	2.888 (30.4)	6.611 (69.6)		
**Eating quick snacks instead of having lunch**				
Yes	596 (26.5)	1.657 (73.5)	0.78 (0.70; 0.86)	<0.001
No	6.599 (32.2)	13.876 (67.8)		
**Type of soft drink consumed^a^**				
Normal	1.834 (32.1)	3.880 (67.9)	1.65 (1.30; 2.08)	<0.001
Zero sugar	110 (20.0)	441 (80.0)		
**Physical activity**				
No	5.736 (35.6)	10.377 (64.4)	1.72 (1.60; 1.85)	<0.001
Yes	1.459 (22.1)	5.156 (77.9)		

^a^The difference in the total number of cases (100.0%) corresponds to number of unknown, blank or unanswered fields on the National Health Survey database.

In the multivariate analysis (Table 2), we found that complete tooth loss in elderly individuals was higher in females (p-value<0.001; prevalence ratio [PR] 1.05; 95%CI 1.04; 1.07), in older elderly individuals (p-value<0.001; PR 1.54; 95%CI 1.43; 1.61), in those with no formal education (p-value<0.001; PR 1.06; 95%CI 1.04; 1.08), in those without dental insurance (p-value<0.001; PR 1.07; 95%CI 1.05; 1.09), in smokers (p-value<0.001; PR 1.04; 95%CI 1.02; 1.06), in individuals who consume soft drinks with high sugar content (p-value<0.001; PR 1.05; 95%CI 1.03; 1.07) and in those who do not undertake physical activity (p-value<0.001; PR 1.05; 95%CI 1.03; 1.06).

**Table 2:** Adjusted prevalence ratios (PR) and adjusted 95% confidence intervals (95%) for complete tooth loss, according to socioeconomic and lifestyle variables. Brazil, 2019 (n=22,728)

	Complete tooth loss present n=7,195	Complete tooth loss absent n=15,533		
Variable	n (%)	n (%)	PR (95%CI)	p-value
Sex				
Female	4,511 (36.0)	8,024 (64.0)	1.05 (1.04; 1.07)	<0.001
Male	2,684 (26.3)	7,509 (73.7)		
**Age** (years)				
60-69	2,917 (23.2)	9,638 (76.8)	1.00	<0.001
70-79	2,736 (38.2)	4,421 (61.8)	1.17 (1.12; 1.25)	
≥80	1,542 (51.1)	1,474 (48.9)	1.54 (1.43; 1.61)	
Schooling				
Illiterate	2,496 (46.4)	2,887 (53.6)	1.06 (1.04; 1.08)	<0.001
Literate	4.699 (27.1)	12,646 (72.9)		
**Dental insurance**				
No	6,910 (33.0)	14,018 (67.0)	1.07 (1.05; 1.09)	
Yes	285 (15.8)	1,515 (84.2)		
**Tobacco smoking**				
Yes	1,002 (37.4)	1,678 (62.6)	1.04 (1.02; 1.06)	<0.001
No	6,193 (30.9)	13,855 (69.1)		
**Type of soft drink consumed^a^**				
Normal	1,834 (32.1)	3,880 (67.9)	1.05 (1.03; 1.07)	<0.001
Zero sugar	110 (20.0)	441 (80.0)		
**Physical activity**				
No	5,736 (35.6)	10,377 (64.4)	1.05 (1.03; 1.06)	<0.001
Yes	1,459 (22.1)	5,156 (77.9)		

^a^The difference in the total number of cases (100.0%) corresponds to number of unknown, blank or unanswered fields on the National Health Survey database.

## Discussion

This study’s null hypothesis was not accepted. Complete tooth loss in the elderly was higher in those with poorer lifestyles. Elderly people who smoke, consume soft drinks with high sugar content and do not undertake regular physical activities lose their teeth completely more frequently. In addition, elderly people with poorer socioeconomic conditions were also more affected by complete tooth loss. Prevalence of complete tooth loss in elderly Brazilians in this study was high. Given the aesthetic, phonation, masticatory performance, nutritional and psychological damage that complete tooth loss can cause, this reality reveals a worrying fact (22,23). High rates of complete tooth loss in the elderly are also found in the literature (2). This panorama highlights the need to identify factors that contribute to tooth loss in order to develop preventive measures.

A limitation of this study is the fact that it was cross-sectional in nature, which may restrict understanding of the causal relationship between lifestyle and complete bimaxillary tooth loss in the elderly. It is therefore recommended that future studies with a longitudinal design be carried out, such as those conducted in institutions for the elderly, where data collection can be more complete, with lower risk of loss of information, as well as enabling collection of complementary data through interviews with family members. Despite this limitation, this study offers results with robust external validity, since the sample used was drawn from the National Health Survey, which is representative of the Brazilian elderly population and covered a wide diversity of socioeconomic, behavioral, and regional conditions. This ensures that the conclusions about factors associated with complete tooth loss in the elderly can be generalized to other elderly populations in Brazil, considering the variation in demographic characteristics and lifestyle among different groups of elderly people. Furthermore, the associated factors identified in this study are common to several populations in similar contexts, increasing the applicability of the findings in other settings. Although the study was conducted in Brazil, the behavioral and socioeconomic patterns found, especially those associated with health habits, are shared by many elderly populations in other developing countries, which increases the relevance of the results for international contexts. Thus, the results of this study can serve as a basis for the formulation of public health policies and prevention strategies in different regions of Brazil, as well as in contexts in the world with similar characteristics.

In this study’s sample, there were losses in some variables analyzed (race/skin color, tooth brushing frequency and type of soft drink consumed) due to this information not being filled in on the database. However, considering the total sample size and the small proportion of these losses, the results of the study are not impaired. The remaining sample continues to be representative, ensuring the robustness of the findings and the generalizability of the conclusions.

The results indicated significant association between complete tooth loss and the female sex. One possible hypothesis for this association is that women, in general, demonstrate greater concern for their health and, consequently, attend health services more regularly. However, due to the historical predominance of mutilating dental practices, they are subjected to a greater number of tooth extractions compared to men. This greater exposure to invasive interventions probably contributes to the high rates of tooth loss among women (24,25). Regarding older elderly individuals, they have been more exposed to the consequences of dental caries and periodontal disease throughout their lives compared to younger individuals. These diseases are the main causes of tooth loss. In addition, older elderly individuals are those who experience greater functional decline, which can compromise the ability to clean the oral cavity properly and consequently lead to a greater number of tooth losses (9,10).

Complete tooth loss was higher in illiterate elderly individuals. These data suggest that illiteracy may play a crucial role in limiting access to information on tooth loss prevention and dental care. This situation makes illiterate elderly individuals more susceptible to tooth loss. Poorer general and oral health conditions are greater in those with lower socioeconomic conditions (26,27). In this study, we found that elderly individuals without dental insurance are more likely to lose their teeth completely. Absence of dental insurance is a strong indicator of lower purchasing power. This indicates that these elderly individuals have unfavorable financial conditions for seeking tooth loss prevention services. Associated with this, absence of dental insurance exposes these elderly individuals to less dental care and deprivation of dental treatments that could prevent tooth loss (28,29).

Regarding lifestyle variables, we found that elderly smokers have a higher frequency of complete tooth loss. Smoking is a significant environmental factor that influences oral pathophysiology. The toxic components present in cigarettes impact the oral microbiota both directly and indirectly through mechanisms such as immunosuppression and oxygen deprivation. These processes culminate in oral diseases, such as periodontitis, which can lead to greater tooth loss. Smokers show signs of consistent gingival bleeding, intense gingival keratinization, and increased probing pocket depth, which contributes to tooth loss (30).

Another lifestyle variable related to tooth loss found in this study was consumption of soft drinks high in sugar. Higher prevalence of tooth loss was found among those who had this habit (31). This is because a diet rich in sugars contributes to a decrease in oral pH due to the action of microorganisms. As a consequence, demineralization and loss of dental tissue are observed (32). Progression of this loss of dental tissue can result in loss of teeth and lead these elderly individuals to a condition of complete tooth loss. Finally, with regard to physical activity, those who do not perform it regularly lose more teeth, according to this study. Physical inactivity is considered one of the main public health problems in the world, being one of the predisposing factors for chronic non-communicable diseases. Lack of activity is related to chronic diseases such as diabetes, hypertension and obesity, contributing to the worsening of oral diseases, such as periodontal disease and consequently to increased tooth loss (18,19). In addition, it is suggested that elderly people who do not undertake physical activities are more fragile and may have motor difficulties in carrying out oral hygiene.

The results of this study indicate that complete tooth loss in the elderly is related to sociodemographic variables, behavioral variables, and variables related to access to oral health care. These findings have important implications for clinical practice and public health policies, suggesting that prevention strategies should be targeted at these specific groups, promoting oral health and healthy habits, in addition to ensuring greater access to dental services. The evidence also opens avenues for future research, such as evaluation of intervention programs that address these risk factors, aiming to reduce the prevalence of tooth loss among the elderly population.

In conclusion, complete bimaxillary tooth loss in the elderly was higher in those with unfavorable socioeconomic conditions and in those with poorer lifestyles, related to smoking habits, drinking soft drinks with high sugar content and not doing physical activity regularly.

## Data Availability

The 2019 National Health Survey database and the analysis codes used in this research are available at: https://www.pns.icict.fiocruz.br/bases-de-dados/.
