# Supplementary material for: Lifestyles associated with complete tooth loss in elderly people in Brazil, 2019
Source: Epidemiol Serv Saude. 2025 May 12;34:e20240614. doi: 10.1590/S2237-96222025v34e20240614.en (PMC12077536; doi:10.1590/S2237-96222025v34e20240614.en)
Supplement: Translation

# Translation: Estilos de vida associados à perda dentária total em idosos no Brasil, 2019

**DOI:** 10.1590/S2237-96222025v34e20240614.pt

**Authors:** Zamir Vidal de Negreiros, Nizyara Costa da Silva, Rafaela Alcindo Silva de Sousa Fé, Mariella Agostinho Gonçalves Lourenço, Rafael Barroso Pazinatto, Ana Estéfanny Alves Cabral, Laércio Almeida de Melo

**Affiliations:** 1 Universidade Federal do Rio Grande do Norte, Departamento de Odontologia, Natal, RN, Brasil; 2 Universidade Federal de Juiz de Fora, Departamento de Odontologia Restauradora, Juiz de Fora, MG, Brasil

**Keywords:** Idoso, Estilo de Vida, Perda de Dente, Fatores Desencadeantes, Estudos Transversais

## Abstract

**Objetivo:**

Este estudo objetivou avaliar os tipos de estilo de vida que podem contribuir para a perda dentária total em idosos.

**Métodos:**

O presente estudo é caracterizado como transversal e de base populacional, tendo como população-alvos indivíduos idosos com 60 anos ou mais. Foi utilizado a base de dados da última edição da Pesquisa Nacional de Saúde realizada no Brasil, em 2019. Inicialmente, foi utilizado o teste qui-quadrado e em seguida as razões de prevalência foram ajustadas a partir do modelo de regressão múltipla de Poisson com o objetivo de identificar associações entre as variáveis.

**Resultados:**

A amostra final analisada foi de 22.728 idosos. A prevalência de edentulismo total foi de 31,7% (intervalo de confiança de 95% [IC95%] 31,1; 32,3). A análise multivariada revelou que essa condição foi maior no sexo feminino (p-valor<0,001; razão de prevalência [RP] 1,05; IC95% 1,04; 1,07), nos idosos mais longevos (p-valor<0,001; RP 1,54; IC95% 1,43; 1,61), naqueles sem escolaridade (p-valor<0,001; RP 1,06; IC95% 1,04; 1,08), nos que não possuem plano odontológico (p-valor<0,001; RP 1,07; IC95% 1,05; 1,09), nos fumantes (p-valor<0,001; RP 1,04; IC95% 1,02; 1,06), nos indivíduos que consomem refrigerantes com alto teor de açúcar (p-valor<0,001; RP 1,05; IC95% 1,03; 1,07) e nos que não praticam atividade física (p-valor<0,001; RP 1,05; IC95% 1,03; 1,06).

**Conclusão:**

Conclui-se, que o edentulismo total foi maior em idosos com condições socioeconômicas desfavoráveis, nos que fumam, nos que consomem refrigerantes com alto teor de açúcar e nos que não realizam atividade física regularmente.

## Introdução

Nas últimas décadas, a prevalência da perda dentária total apresentou diminuição em populações mais jovens, entretanto continua prevalente em idosos (1). As doenças bucais ainda afetam muitos idosos, sendo a cárie dentária e a progressão da doença periodontal os principais fatores que influenciam a perda dentária. Esta pode contribuir com problemas funcionais, como a mastigação e a fonética. Problemas estéticos e sociais podem ser observados, afetando a saúde e a qualidade de vida, principalmente quando o edentulismo é total (2). O edentulismo possui etiologia multifatorial e envolve fatores biológicos, físicos, econômicos, culturais, sociais e até mesmo comportamentais (3-6). 

Os resultados mais frequentes encontrados na literatura sobre os fatores associados ao edentulismo total ou parcial são aqueles relacionados à saúde bucal (7). É importante destacar que fatores sistêmicos, como doenças crônicas não transmissíveis, fragilidade e incapacidade motora, podem ser variáveis importantes no declínio da saúde bucal, que por consequência podem contribuir com a perda dentária (8-11). Hábitos como o tabagismo e o consumo de bebidas alcoólicas e estilos de vida precários também podem influenciar na piora da saúde bucal. Essas variáveis não são incluídas na maioria dos estudos sobre perda dentária.

A nicotina presente no tabaco pode contribuir com a formação de biofilme cariogênico, assim como as alterações na composição e na quantidade de saliva (12-15). Além de influenciar na incidência de cárie dentária, o tabaco é fator de risco quando se trata de doença periodontal. Os fumantes apresentam maior inflamação dos tecidos periodontais, o que contribui para o risco aumentado ao desenvolvimento e ao agravamento da periodontite (16,17). 

Alterações salivares e danificações de tecidos moles na boca podem ser evidentes naqueles indivíduos que possuem o hábito de consumir bebida alcoólica. Apesar de não ter uma relação direta com o edentulismo total, estilos de vida não saudáveis, como a ausência de práticas de exercício físico, contribuem para a deterioração funcional e sistêmica do idoso e podem influenciar na saúde bucal (18,19).

Diante do envelhecimento acelerado da população mundial e das consequências relacionadas à perda dentária, este estudo objetivou avaliar os tipos de estilo de vida que podem contribuir para a perda dentária total em idosos. A hipótese nula do presente estudo é de que o edentulismo total não são maiores em idosos com piores hábitos e estilos de vida não saudáveis.

## Métodos

### Delineamento do estudo

Trata-se de estudo transversal, observacional e de base populacional. Para que este estudo fosse produzido, foi utilizada a base de dados da Pesquisa Nacional de Saúde realizada no Brasil em 2019. 

### Contexto

A Pesquisa Nacional de Saúde de 2019 foi realizada pelo Instituto Brasileiro de Geografia e Estatística em parceria com o Ministério da Saúde. Essa pesquisa objetivou fornecer dados atualizados sobre a saúde da população brasileira. A pesquisa teve como população-alvo pessoas com 15 anos ou mais e abordou aspectos como condições de saúde, estilo de vida, acesso a serviços de saúde, morbidade, uso de medicamentos e saúde mental, além de traçar o panorama das condições de vida da população, com ênfase nas desigualdades regional e socioeconômica. A coleta de dados ocorreu entre agosto de 2019 e março de 2020.

### Participantes

Neste estudo, foram analisados indivíduos com 60 anos ou mais que apresentaram informações sobre a presença ou não da perda dentária total bimaxilar. A amostra da Pesquisa Nacional de Saúde é representativa da população idosa do Brasil. Detalhes sobre o cálculo amostral podem ser encontrados na seção de metodologia da Pesquisa Nacional de Saúde (20).

### Variáveis independentes

As variáveis independentes, as quais possuem possível influência no desfecho para o edentulismo total, foram coletadas por meio do questionário utilizado na Pesquisa Nacional de Saúde. Tais variáveis são: sexo, idade, raça/cor da pele, escolaridade, frequência de escovação, plano odontológico, tabagismo, consumo de álcool, prática de exercício físico, consumo de biscoitos doces ou chocolates, substituição de almoço por lanches rápidos e tipo de refrigerante consumido.

### Edentulismo total

A identificação dos indivíduos que apresentavam edentulismo total superior e inferior foi feita pela análise do questionário aplicado na Pesquisa Nacional de Saúde por meio das seguintes perguntas: “Lembrando-se dos seus dentes permanentes de cima, o(a) senhor(a) perdeu algum?” e “Lembrando-se dos seus dentes permanentes de baixo, o(a) senhor(a) perdeu algum?”. As perguntas em questão poderiam ter como respostas: “Não”, “Sim, perdi XX dentes”, em que “XX” representava a quantidade de dentes perdidos, “Sim, perdi todos os dentes de cima” e “Sim, perdi todos os dentes de baixo”. A variável “Edentulismo total” foi categorizada em “presente”, quando todos os dentes superiores e inferiores foram perdidos, ou “ausente”, quando não houve a perda total dos elementos dentários.

### Fonte de dados e mensuração

Este trabalho fez uso de dados secundários da Pesquisa Nacional de Saúde de 2019, conduzida no Brasil. Os dados podem ser acessados na seguinte fonte: https://www.pns.icict.fiocruz.br/bases-de-dados/ (21).

### Controle de viés

O controle de viés neste estudo foi fundamentado na abordagem de amostragem da Pesquisa Nacional de Saúde, a qual considerou as diferentes probabilidades de seleção e as características do desenho amostral complexo. Foram atribuídos pesos amostrais tanto aos domicílios quanto aos indivíduos selecionados. O peso final foi determinado pelo inverso das probabilidades de seleção em cada etapa do plano amostral, com ajustes para não resposta e correções dos totais populacionais. Devido à origem da amostra na pesquisa por conglomerados, foi utilizado um software de análise estatística especializado, que levou em conta os efeitos da estratificação e da agregação dos dados na estimação dos indicadores e na determinação das respectivas medidas de precisão.

### Tamanho do estudo

A Pesquisa Nacional de Saúde foi conduzida em todo o Brasil. Abrangeram-se todas as macrorregiões, incluindo zonas urbanas e rurais, e foram considerados domicílios nas capitais e regiões metropolitanas. Com sua abrangência e metodologia rigorosa, a pesquisa fornece o panorama completo das condições de saúde da população brasileira.

### Métodos estatísticos

A análise dos dados foi realizada por meio do programa estatístico Statistical Package for the Social Sciences, versão 22.0. As distribuições de frequência das variáveis do estudo foram realizadas para criar as tabelas. Foi utilizado o teste qui-quadrado com nível de confiança de 95% para verificar a associação entre a perda total de elementos dentários e as variáveis independentes. Diante dos resultados desse teste, as variáveis que obtiveram valor de p-valor<0,200 foram submetidas ao teste qui-quadrado entre elas para testar a multicolinearidade. Variáveis altamente relacionadas entre si (multicolineares) não foram incluídas no modelo de ajuste. Em consequência do tamanho amostral do estudo, as variáveis foram consideradas multicolineares quando foi obtido p-valor<0,001. As razões de prevalência ajustadas foram estimadas através da regressão múltipla de Poisson. Em todos os testes, os dados foram ponderados considerando sempre o efeito de plano amostral, os pesos pós-estratificação e as taxas de não respostas. Em relação à regressão múltipla de Poisson, foi utilizado também o nível de confiança de 95%.

## Resultados

A amostra deste estudo foi de 22.728 idosos, com idade média de 70,0 anos (±7,8), variando de 60-107 anos. A análise de frequência dos resultados evidenciou que a maioria dos idosos edêntulos totais que moram no Brasil é do sexo feminino (62,7%), com 60-69 anos (40,5%), de cor branca (40,4%), alfabetizados (65,3%), sem plano odontológico (96,0%), que não fazem uso de bebida alcoólica (84,7%), não fumam (86,1%), escovam os dentes pelo menos uma vez ao dia (99,1%), não consomem biscoitos doces ou chocolates na semana (59,9%), não substituem almoço por lanches rápidos em algum dia da semana (91,7%), consomem refrigerante com açúcar (94,3%) e não realizam algum tipo de atividade física (79,7%).

A prevalência de idosos edêntulos totais foi de 31,7% (intervalo de confiança de 95% [IC95%] 31,1; 32,3. A frequência das variáveis independentes (variáveis socioeconômicas e de estilo de vida), assim como suas associações com o edentulismo total por meio da análise univariada estão apresentadas na Tabela 1. Como resultado desta primeira análise e da verificação da multicolinearidade entre as variáveis, a “raça/cor de pele”, “bebida alcoólica”, “frequência de escovação”, “consumo de biscoitos doces ou chocolates” e “substituição de almoço por lanches rápidos” não foram incluídos no modelo de ajuste da regressão múltipla de Poisson devido à forte associação com as outras variáveis independentes.

**Tabela 1:** Razões de prevalências (RP) brutas e intervalos de confiança de 95% (IC95%) brutos do edentulismo total pelas variáveis socioeconômicas e de estilo de vida. Brasil, 2019 (n=22.728)

	Edentulismo total presente n=7.195	Edentulismo total ausente n=15.533		
Variável	n (%)	n (%)	RP (IC95%)	p-valor
Sexo				
Feminino	4.511 (36,0)	8.024 (64,0)	1,40 (1,32; 1,48)	<0,001
Masculino	2.684 (26,3)	7.509 (73,7)		
**Idade** (anos)				
60-69	2.917 (23,2)	9.638 (76,8)	1,00	<0,001
70-79	2.736 (38,2)	4.421 (61,8)	1,26 (1,23; 1,31)	
≥80	1.542 (51,1)	1.474 (48,9)	1,62 (1,53; 1,72)	
**Raça/cor da pele^a^**				
Branco	2.906 (29,4)	6.995 (70,6)	1,00	<0,001
Pardo	3.399 (34,0)	6.602 (66,0)	1,07 (1,04; 1,11)	
Negro	779 (31,7)	1.676 (68,3)	1,06 (1,01; 1,12)	
Outros	110 (29,8)	259 (70,2)	0,96 (0,87; 1,05)	
Escolaridade				
Não alfabetizado	2.496 (46,4)	2.887 (53,6)	1,75 (1,65; 1,86)	<0,001
Alfabetizado	4.699 (27,1)	12.646 (72,9)		
**Plano odontológico**				
Não	6.910 (33,0)	14.018 (67,0)	2,09 (1,76; 2,48)	
Sim	285 (15,8)	1.515 (84,2)		
**Bebida alcoólica**				
Sim	1.102 (19,6)	4.530 (80,4)	0,51 (0,46; 0,56)	<0,001
Não	6.093 (35,6)	11.003 (64,4)		
Fumo				
Sim	1.002 (37,4)	1.678 (62,6)	1,24 (1,14; 1,35)	<0,001
Não	6.193 (30,9)	13.855 (69,1)		
**Frequência de escovação^a^**				
Não escova todos os dias	61 (48,0)	66 (52,0)	1,32 (1,00; 1,75)	0,074
Pelo menos uma vez ao dia	6.375 (29,7)	15.139 (70,4)		
**Consumo de biscoitos doces ou chocolates**				
Sim	4.307 (32,6)	8.922 (67,4)	1,10 (1,04; 1,17)	<0,001
Não	2.888 (30,4)	6.611 (69,6)		
**Substituição de almoço por lanches rápidos**				
Sim	596 (26,5)	1.657 (73,5)	0,78 (0,70; 0,86)	<0,001
Não	6.599 (32,2)	13.876 (67,8)		
**Tipo de refrigerante consumido^a^**				
Normal	1.834 (32,1)	3.880 (67,9)	1,65 (1,30; 2,08)	<0,001
Zero açúcar	110 (20,0)	441 (80,0)		
**Atividade física**				
Não	5.736 (35,6)	10.377 (64,4)	1,72 (1,60; 1,85)	<0,001
Sim	1.459 (22,1)	5.156 (77,9)		

^a^A diferença para o número total de casos (100,0%) corresponde ao número de casos ignorados, em branco ou com preenchimento ausente no banco de dados da Pesquisa Nacional de Saúde.

Na análise multivariada (Tabela 2), verificou-se que o edentulismo total em idosos foi maior no sexo feminino (p-valor<0,001; razão de prevalência [RP] 1,05; IC95% 1,04; 1,07), nos idosos mais longevos (p-valor<0,001; RP 1,54; IC95% 1,43; 1,61), naqueles sem escolaridade (p-valor<0,001; RP 1,06; IC95% 1,04; 1,08), nos que não possuem plano odontológico (p-valor<0,001; RP 1,07; IC95% 1,05; 1,09), nos fumantes (p-valor<0,001; RP 1,04; IC95% 1,02; 1,06), nos indivíduos que consomem refrigerantes com alto teor de açúcar (p-valor<0,001; RP 1,05; IC95% 1,03; 1,07) e nos que não praticam atividade física (p-valor<0,001; RP 1,05; IC95% 1,03; 1,06).

**Tabela 2:** Razões de prevalências ajustadas (RP) e intervalos de confiança de 95% ajustados (IC95%) do edentulismo total pelas variáveis socioeconômicas e de estilo de vida. Brasil, 2019 (n=22.728)

	Edentulismo total presente n=7.195	Edentulismo total ausente n=15.533		
Variável	n (%)	n (%)	RP (IC95%)	p-valor
Sexo				
Feminino	4.511 (36,0)	8.024 (64,0)	1,05 (1,04; 1,07)	<0,001
Masculino	2.684 (26,3)	7.509 (73,7)		
**Idade** (anos)				
60-69	2.917 (23,2)	9.638 (76,8)	1,00	<0,001
70-79	2.736 (38,2)	4.421 (61,8)	1,17 (1,12; 1,25)	
≥80	1.542 (51,1)	1.474 (48,9)	1,54 (1,43; 1,61)	
Escolaridade				
Não alfabetizado	2.496 (46,4)	2.887 (53,6)	1,06 (1,04; 1,08)	<0,001
Alfabetizado	4.699 (27,1)	12.646 (72,9)		
**Plano odontológico**				
Não	6.910 (33,0)	14.018 (67,0)	1,07 (1,05; 1,09)	
Sim	285 (15,8)	1.515 (84,2)		
Fumo				
Sim	1.002 (37,4)	1.678 (62,6)	1,04 (1,02; 1,06)	<0,001
Não	6.193 (30,9)	13.855 (69,1)		
**Tipo de refrigerante consumido^a^**				
Normal	1.834 (32,1)	3.880 (67,9)	1,05 (1,03; 1,07)	<0,001
Zero açúcar	110 (20,0)	441 (80,0)		
**Atividade física**				
Não	5.736 (35,6)	10.377 (64,4)	1,05 (1,03; 1,06)	<0,001
Sim	1.459 (22,1)	5.156 (77,9)		

^a^A diferença para o número total de casos (100,0%) corresponde ao número de casos ignorados, em branco ou com preenchimento ausente no banco de dados da Pesquisa Nacional de Saúde.

## Discussão

A hipótese nula do presente estudo não foi aceita. O edentulismo total em idosos foi maior naqueles com piores estilos de vida. Aqueles idosos que possuem o hábito de fumar, que consomem refrigerantes com alto teor de açúcar e os que não praticam de forma regular atividades físicas, perdem totalmente seus dentes com mais frequência. Além disso, os idosos com piores condições socioeconômicas também foram mais acometidos com o edentulismo total. A prevalência de edentulismo total em idosos brasileiros neste estudo foi elevada. Diante dos prejuízos estéticos, de fonação, de performance mastigatória, nutricional e psicológico que a perda dentária total pode trazer, essa realidade mostra um dado preocupante (22,23). Altas taxas de edentulismo total em idosos também são observados na literatura (2). Esse panorama evidencia a necessidade de identificar os fatores que contribuem para a perda dentária a fim de elaborar medidas preventivas.

Este estudo apresenta como limitação o fato de ser de natureza transversal, o que pode restringir a compreensão da relação causal entre o estilo de vida e o edentulismo total bimaxilar em idosos. Recomenda-se, portanto, a realização de estudos futuros com delineamento longitudinal, como aqueles conduzidos em instituições para idosos, onde a coleta de dados pode ser mais completa, com menor risco de perda de informações e permitindo a obtenção de dados complementares por meio de entrevistas com familiares. Apesar dessa limitação, este trabalho oferece uma validade externa dos resultados robusta, uma vez que a amostra utilizada foi extraída da Pesquisa Nacional de Saúde, que é representativa da população idosa brasileira e abrangeu uma ampla diversidade de condições socioeconômicas, comportamentais e regionais. Isso garante que as conclusões sobre os fatores associados ao edentulismo total em idosos possam ser generalizadas para outras populações idosas no Brasil, considerando a variação nas características demográficas e no estilo de vida entre diferentes grupos de idosos. Além disso, os fatores associados identificados neste estudo são comuns a várias populações em contextos semelhantes, aumentando a aplicabilidade dos achados em outros cenários. Embora o estudo tenha sido realizado no Brasil, os padrões comportamentais e socioeconômicos observados, especialmente os associados a hábitos de saúde, são compartilhados por muitas populações idosas em outros países em desenvolvimento, o que amplia a relevância dos resultados para contextos internacionais. Dessa forma, os resultados deste estudo podem servir como base para a formulação de políticas de saúde pública e estratégias de prevenção em diferentes regiões do Brasil, bem como em contextos globais com características semelhantes.

Na amostra deste estudo, houve perdas em algumas variáveis analisadas (raça/cor de pele, frequência de escovação e tipo de refrigerante consumido) devido ao não preenchimento do banco de dados para essas informações. Entretanto, considerando o tamanho total da amostra e a proporção reduzida dessas perdas, não se observa comprometimento nos resultados do estudo. A amostra remanescente permanece representativa, garantindo a robustez dos achados e a generalização das conclusões.

Os resultados indicaram uma associação significativa entre o edentulismo total e o sexo feminino. Uma possível hipótese para essa associação está no fato que as mulheres, em geral, demostram uma maior preocupação com a saúde e, consequentemente, frequentam serviços de saúde com maior regularidade. Contudo, devido a predominância histórica de práticas odontológicas mutiladoras, elas são submetidas a um número maior de extrações dos elementos dentários em comparação aos homens. Provavelmente, essa maior exposição a intervenções invasivas contribui para os índices elevados de edentulismo entre as mulheres (24,25). No que se refere aos idosos mais velhos, esses indivíduos ao longo da vida estão mais expostos às consequências da cárie dentária e doença periodontal em relação aos mais jovens. Essas doenças são as principais causas da perda dentária. Além disso, idosos mais velhos são os que possuem maior declínio funcional, o que pode comprometer a capacidade de higienizar a cavidade bucal de forma satisfatória e por consequência levar a um maior número de perdas dentárias (9,10). 

O edentulismo total foi maior em idosos analfabetos. Esses dados sugerem que o analfabetismo pode desempenhar um papel crucial na limitação do acesso às informações sobre prevenção do edentulismo e cuidados odontológicos. Esta situação torna os idosos analfabetos mais susceptíveis ao edentulismo. Piores condições de saúde geral e bucal são maiores naqueles com piores condições socioeconômicas (26,27). Neste estudo, verificou-se que os idosos sem plano odontológico estão sujeitos a perderem mais os seus dentes totalmente. A ausência de plano odontológico é um forte indicador de um menor poder aquisitivo. Isso indica que esses idosos possuem condições financeiras desfavoráveis para buscar serviços de prevenção a perda dentária. Associado a isso, a ausência de plano odontológico expõe esses idosos a menores cuidados odontológicos e a privação de tratamentos dentários que poderiam evitar a perda dentária (28,29).

Em relação as variáveis de estilo de vida, observou-se que idosos fumantes apresentam maior frequência de edentulismo total. O tabagismo é um fator ambiental significante que influencia a fisiopatologia oral. Os componentes tóxicos presentes no cigarro impactam a microbiota oral tanto direta quanto indiretamente através de mecanismos como imunossupressão e privação de oxigênio. Esses processos culminam em doenças orais, como periodontite, que podem levar a uma maior perda dentária. Os fumantes apresentam sinais de sangramento gengival consistente, ceratinização gengival intensa e aumento da profundidade da bolsa de sondagem, o que contribui para a perda dentária (30).

Outra variável de estilo de vida relacionada ao edentulismo verificada neste estudo foi o consumo de refrigerante com alto teor de açúcar. Uma maior prevalência de perda dentária foi maior em quem possui esse hábito (31). Isso se deve ao fato de uma dieta rica em açucares contribuir para a diminuição do pH bucal devido à ação de microrganismos. Como consequência, uma desmineralização e perda de tecido dentário são observados

(32). A progressão dessa perda de tecido dentário pode evoluir para a perda do elemento dentário e levar esses idosos a uma condição de edentulismo total. Por fim, no que se refere a atividade física, os que não as realizam com regularidade perdem mais seus elementos dentários de acordo com este estudo. A inatividade física é considerada um dos principais problemas de saúde pública no mundo, sendo um dos fatores predisponentes para doenças crônicas não transmissíveis. A falta de atividade está relacionada às doenças crônicas como diabetes, hipertensão e obesidade, contribuindo com o agravamento de doenças bucais, como a doença periodontal e consequentemente com o aumento de perdas dentárias (18,19). Além disso, sugere-se que os idosos que não realizam atividades físicas são mais frágeis e podem ter dificuldade motora para a higiene bucal.

Os resultados deste estudo indicam que o edentulismo total em idosos possui relação com variáveis sociodemográficas, variáveis comportamentais e variáveis de acesso à saúde bucal. Essas descobertas têm importantes implicações para a prática clínica e políticas de saúde pública, sugerindo que estratégias de prevenção devem ser direcionadas a esses grupos específicos, promovendo a saúde bucal e hábitos saudáveis, além de garantir maior acesso aos serviços odontológicos. As evidências também abrem caminhos para futuras pesquisas, como a avaliação de programas de intervenção que abordem esses fatores de risco, visando reduzir a prevalência do edentulismo na população idosa.

Como conclusão, o edentulismo total bimaxilar em idosos foi maior naqueles com condições socioeconômicas desfavoráveis e nos que possuem piores estilos de vida, relacionados ao hábito de fumar, beber refrigerantes com alto teor de açúcar e a não realização de atividade física de forma regular.

## Data Availability

O banco de dados utilizado da Pesquisa Nacional de Saúde de 2019 e os códigos de análise utilizados na pesquisa estão disponíveis em: https://www.pns.icict.fiocruz.br/bases-de-dados/
